# Marginal fit of 3-unit implant-supported fixed partial dentures: Influence of pattern fabrication method and repeated porcelain firings

**DOI:** 10.1371/journal.pone.0301799

**Published:** 2024-04-16

**Authors:** Rashin Giti, Pardis Farrahi

**Affiliations:** 1 Oral and Dental Diseases Research Center, Faculty of Dentistry, Shiraz University of Medical Sciences, Shiraz, Fars, Iran; 2 Department of Prosthodontics, Faculty of Dentistry, Shiraz University of Medical Sciences, Shiraz, Fars, Iran; 3 Student Research Committee, Faculty of Dentistry, Shiraz University of Medical Sciences, Shiraz, Fars, Iran; Yerevan State Medical University Named after Mkhitar Heratsi, ARMENIA

## Abstract

**Background:**

Marginal fit significantly impacts the long-term success of dental restorations. Different pattern fabrication methods, including hand-waxing, milling, or 3D printing, may affect restorations accuracy. The effect of porcelain firing cycles on the marginal fit of metal-ceramic restorations remains controversial, with conflicting findings across studies.

**Purpose:**

The aim was to evaluate the potential effects of multiple porcelain firings (3, 5, 7 cycles) as well as pattern fabrication method (conventional hand-waxing, milling, and 3D printing) on the marginal adaptation of 3-unit implant-supported metal-ceramic fixed partial dentures. It was hypothesized that neither the wax pattern fabrication method nor repeated ceramic firings would significantly affect the marginal adaptation of metal-ceramic crowns.

**Methods:**

In this in-vitro study, 30 Cobalt-Chromium alloy frameworks were fabricated based on pattern made through three techniques: conventional hand-waxing, CAD-CAM milling, and CAD-CAM 3D printing (*n* = 10 per group). Sixteen locations were marked on each abutment to measure the vertical marginal gap at four stages: before porcelain veneering and after 3, 5, and 7 firing cycles. The vertical marginal gap was measured using direct microscopic technique at ×80 magnification. Mean vertical marginal gap values were calculated and two-way ANOVA and Tukey’s post hoc tests were used for inter-group comparisons (α = 0.05).

**Results:**

The 3D printing group showed significantly lower (*P*<0.001) mean vertical marginal gaps (60–76 μm) compared to the milling (77–115 μm) and conventional hand-waxing (102–110 μm) groups. The milling group exhibited a significant vertical gap increase after 3 firing cycles (*P*<0.001); while the conventional (*P* = 0.429) and 3D printing groups (*P* = 0.501) showed no significant changes after 7 firing cycles. Notably, the vertical marginal gap in all groups remained below the clinically acceptable threshold of 120 μm.

**Conclusion:**

CAD-CAM 3D printing provided superior marginal fit compared to CAD-CAM milling and conventional hand-wax pattern fabrication methods. The impact of porcelain firing on the mean marginal gap was significant only in the milling group. All three fabrication techniques yielded clinically acceptable vertical marginal adaptation after repeated firings. Additive manufacturing holds promise to produce precise implant-supported prostheses.

## 1. Introduction

Dentists have long sought to create natural-looking and functionally optimal dental restorations [1]. Long-term success of dental restorations relies on several factors, including proper fit [1–3]. In implant-supported prostheses, poor fit can induce internal stresses on implant components and bone, promote bacterial colonization, and consequently lead to mechanical and biological failures, including screw loosening, crown debonding, fracture of different implant parts, peri-implantitis, and potential loss of osseointegration [4–12]. Although there is no consensus on the maximum clinically acceptable marginal misfit, many studies suggest 120 μm as the maximum limit in single-tooth restorations [13–18]. Marginal misfit, also known as marginal discrepancy, refers to the gap measured along the long axis between the finish line of the tooth preparation and the margin of the restoration [19].

The wax pattern fabrication method is one of the key factors affecting the marginal fit of dental restorations [20, 21]. Achieving acceptable marginal adaptation requires high precision at the initial pattern fabrication stage, as any distortions or defects in the pattern will impart inaccuracies in the final metal coping and prosthesis [21–23]. The main techniques for fabricating wax patterns are conventional hand-waxing, subtractive milling, and additive manufacturing [15]. Conventional hand-waxing, used for decades in fabricating fixed restorations, has shown promising longevity [24]. However, this multiple-steps method carries risks of inaccuracy due to material properties, laboratory procedures, and technician errors [25].

Modern computer-aided design-computer-aided manufacturing (CAD-CAM) technologies were developed to address the limitations of conventional hand waxing by reducing human errors, simplifying processes, increasing production, and fabricating standardized dental prosthetics [14, 26, 27]. CAD-CAM techniques can be categorized as either subtractive, where a solid block is milled, or additive, where the product is built through incremental layers [15, 26]. Although subtractive milling offers significant advantages in production speed and volume compared to the hand wax technique, its reliance on the bur size for accuracy limits its ability to produce fine details and complex geometries, while also generating high material waste [17, 28, 29]. Additive CAD-CAM 3D printing method can minimize material waste and create complex geometries and undercuts not achievable through subtractive technique. However, factors such as layer thickness and post-processing of the structures may impact precision [27, 30–33].

Porcelain-fused-to-metal crowns combine the strength and precision of metal substructures with the aesthetic properties of layered porcelain [34–36]. Meeting clinical and aesthetic standards for porcelain-fused-to-metal crowns necessitates multiple porcelain firings in a vacuum at temperatures ranging from 950°C to 1010°C. These high temperature firings carry a risk of framework distortion that can ultimately jeopardize the marginal fit [34, 37, 38].

Numerous studies have investigated the impacts of porcelain firing cycles on the marginal fit of porcelain-fused-to-metal restorations using various alloy types, margin designs, and manufacturing methods [5, 9, 37–43]. Porcelain firing has been widely reported to increase the marginal gap [5, 40, 43–51]. However, findings are conflicting regarding which firing stages cause the greatest distortion. Contradictory evidence suggests that repeated firings do not necessarily result in a significant increase in the marginal gap [37–39, 52, 53], and there are indications that they could potentially enhance the fit [41].

Several studies have pinpointed the degassing firing preceding porcelain application as the primary cause of marginal discrepancy [19, 35, 36, 54–57]. Buchanan et al. [19] and Campbell et al. [55] reported that the degassing firing step induced the highest marginal opening. Additionally, Shillingburg et al. [44] found that the most significant marginal changes occurred during the porcelain addition stages. Shokry et al. [58] concluded that both porcelain firing and metal selection affect the marginal fit of titanium-based porcelain-fused-to-metal restorations, with the opaque layer firing causing the highest discrepancies. Quante et al. [59] reported only an insignificant marginal gap increase in selective laser melting Co-Cr crowns after laboratory ceramic firing. Zeng et al. [39] discovered no significant changes in marginal fit for up to 7 repeated firings of both selective laser melting and conventional cast copings.

Meanwhile, additional studies have discovered that the impact of porcelain firing on marginal fit varies based on the restoration fabrication method [9, 42]. Onöral et al. [42] found that repeated firings affected the fit of metal-ceramic restorations produced through soft alloy milling, hard alloy milling, and selective laser sintering, but did not significantly influence the fit of the conventional group. As revealed in the literature, there remains considerable controversy surrounding the extent of marginal distortion caused by porcelain firing cycles.

No study has compared the marginal fit of 3-unit copings produced based on patterns fabricated through milling, 3D printing, and conventional hand wax methods, before and after repeated porcelain firing cycles. Given the inadequacy and controversy surrounding the existing research findings, the primary aim of this study was to investigate the influence of multiple porcelain firings (3, 5, and 7 cycles) on the marginal discrepancy of 3-unit implant-supported fixed partial dentures, using different pattern production methods: conventional hand wax, CAD-CAM milling, and CAD-CAM 3D printing. The first null hypothesis posited that the wax pattern fabrication method has no significant effect on the marginal adaptation of frameworks. The second null hypothesis proposed that the marginal adaptation of metal-ceramic crowns is not significantly affected by repeated ceramic firings.

## 2. Materials and methods

This *in-vitro* experimental study was ethically approved by the Ethics Committee of Shiraz University of Medical Sciences (IR.SUMS.DENTAL.REC.1401.115).

### 2.1. Sample preparation

**Fig 1:**
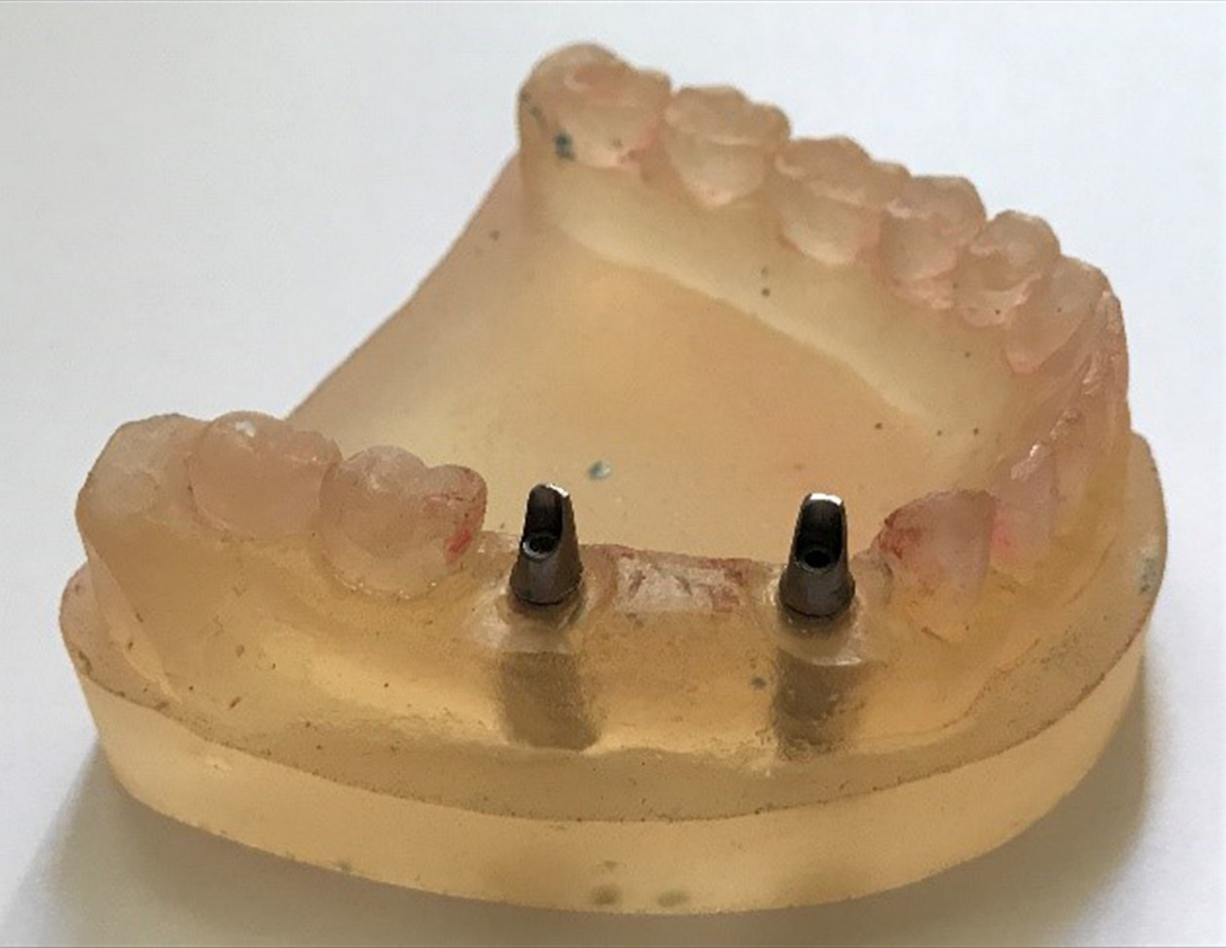
Inserting fixture analogs and straight abutments in the Dentiform.

The study was conducted using a mandibular Dentiform with anatomically accurate tooth morphology, fabricated with heat-curing resin (ACRON No. 5, GC, Tokyo, Japan). Two fixture analogs (Fixture Lab analog, DIO UF(II) Dental Implant System, South Korea) were parallelly inserted using parallelometer (Paraskop M; Bego GmbH, Bremen, Germany), with their axes perpendicular to the horizontal plane. They were positioned at the first premolar and first molar locations to allow the simulation of 3-unit bridges commonly required in the posterior region (**Fig 1**). Straight abutments (Solid abutment, DIO UF(II) Dental Implant System, South Korea) with 6° of convergence and 6 mm length were connected to the fixture analogs per the manufacturer’s instructions. To enhance scanning precision, the Dentiform was sprayed with a scan spray (Arti-Scan CAD/CAM Spray; Dr. Jean Bausch GmbH & Co KG, Köln, Germany) according to the manufacturer’s instruction, and scanned by using a 3D laser scanner (UP400; UP3D, Shenzhen, China) linked to CAD software (Exocad DentalCAD 2.3 Matera; Exocad GmbH, Darmstadt, Germany).

### 2.2. Pattern fabrication

Thirty resins or wax patterns were prepared through the conventional, milling, and 3D printing methods (*n* = 10 per group). The sample size was selected according to a previous study [37] and considering a 5% significance level, power (1-β) of 80%, effect size = 150%, and one-by-one ratio (r) = 1, using the formula $n=\frac{\frac{1+r}{r}s^{2}{\left(z_{1-\alpha /2}+z_{1-\beta }\right)}^{2}}{{\left(d\right)}^{2}}$, the sample size was calculated to be 10 per subgroup.

**Fig 2:**
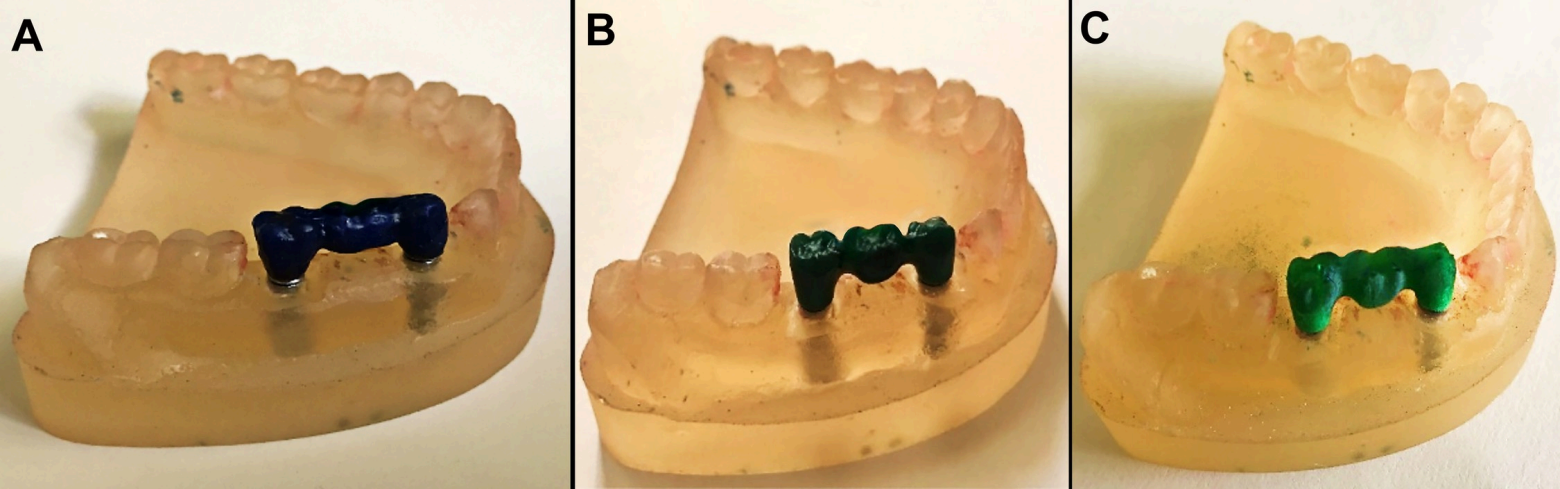
Patterns fabricated through different methods: A) conventional B) milling C) 3D printing.

Patterns with 0.5-mm thickness, 7-mm height, 5-mm mesiodistal length, 5-mm buccolingual width, 40-μm occlusal/axial cement space, and no cement space at the margin were designed for the CAD-CAM milling and 3D printing groups using CAD software (Exocad DentalCAD 2.3 Matera; Exocad GmbH, Darmstadt, Germany). An experienced dental technician digitally modeled Proper emergence profiles to match the dies. In the milling group, a milling unit (CORiTEC 350i series; imes-icore GmbH, Eiterfeld, Germany) was used to mill the wax patterns out of wax blocks (Dental Wax Disc Block; Wieland, China). In the 3D printing group, a 3D printer (Guider 3 Plus; FlashForge, Zhejiang, China) was used to fabricate the wax patterns (Print2Cast Wax Filament; MachinableWax.com, Michigan, United States) based on the same stereolithography input (**Fig 2**).

To fabricate conventional patterns in the laboratory, two layers of die spacer (Pico-fit; Renfert GmbH, Hilzingen, Germany) were directly applied on each abutment wall, 0.5 mm from the margin with a total thickness of 40 μm according to the manufacturer’s instruction. This was done to create cement space that matches the designed cement space thickness in the CAD/CAM groups. Abutments were additionally coated with a thin layer of separating medium (Picosep; Renfert GmbH, Hilzingen, Germany). A silicon index created from the initial CAD-CAM pattern served as a guide to standardize the thickness and contour of the conventional wax patterns. The conventional wax-up procedure was performed using the dip wax technique with inlay wax (Polywax; Bilkimya, Izmir, Turkey) shaped according to the index using electric waxing instruments (Waxelectric II, Renfert GmbH, Hilzingen, Germany). Coping thickness of 0.5 mm was confirmed using a thickness gauge (POCO 2N; Kroeplin, Schluchtern, Germany).

### 2.3. Casting procedures

The wax sprues were attached and the patterns were invested in a phosphate-bonded investment material (Hinrivest RP, ERNST HINRICHS Dental GmbH, Goslar, Germany) using a 20 mL:100 g liquid-to-powder ratio per the manufacturer’s instructions to minimize wax pattern contraction. Wax elimination occurred in a preheated furnace (Magma, Renfert GmbH, Hilzingen, Germany) at 950°C for 40 minutes to ensure complete wax burnout. Cobalt-chromium alloy copings (DAMCAST NB, China) were then cast using a casting machine (Ducatron casting machine; KFP Dental Co., Tehran, Iran). Following the devesting process, the sprues were removed with separating disks (Dentaurum GmbH & Co KG, Ispringen, Germany).

The copings were assessed for errors, including porosities and nodules. The internal surface was inspected for interferences that could prevent full seating of frameworks using a disclosing agent (Occlude indicator, Pascal International Inc., Seattle, Washington, United States) adjusted with a low-speed rotary instrument. Before porcelain application, the outer cameo surfaces were airborne-particle abraded with 110-μm aluminum oxide (Basic Classic, Renfert GmbH, Germany) for 10 seconds at 0.2 Mpa pressure from approximately a 20-mm distance. The Copings were then cleaned ultrasonically for 15 minutes in 95% methyl alcohol and dried with oil-free compressed air (Elmasonic S100H, Elma GmbH & Co KG, Germany).

### 2.4. Porcelain veneering and firing

**Fig 3:**
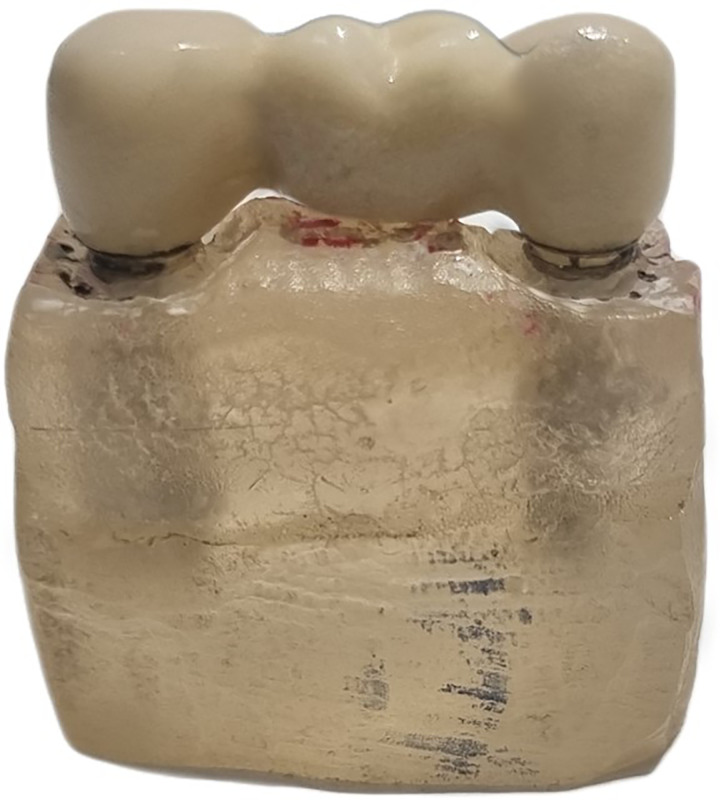
Veneered restorations after glazing.

For porcelain veneering, the copings were initially placed in a dental porcelain furnace (Programat EP 5000/G2, Ivoclar Vivadent AG, Liechtenstein) for oxide layer formation. Opaque and veneering porcelain (VITA Zahnfabrik, Germany) were applied following the manufacturer’s recommendations, using a silicone mold to standardize ceramic thickness. The repeated porcelain firing sequence comprised initial oxidation, opaque firing, dentin/body firing, enamel/incisal firing, laboratory correction firing, clinical correction firing, and glaze firing. The specific temperature, duration, heating rate, and vacuum parameters for each firing stage are presented in Table 1. While 5–6 firing cycles are usually sufficient for metal-ceramic restoration, some clinical scenarios require additional correction firings for shape and color adjustments. To evaluate the potential impacts of these additional firing cycles, this study incorporated 7 total porcelain firings. The vertical marginal discrepancy was measured at four stages: pre-veneering, after the third (body) firing, after the fifth (laboratory correction) firing, and after the seventh (glaze) firing (**Fig 3**). All laboratory procedures were performed by a single skilled technician using standardized protocols.

**Table 1:** Firing parameters of ceramic veneering.

Firing purpose	T (°C)	S [5]	t↑ (°C/min)	H [5]	V_1_ (°C)	V_2_ (°C)
Initial oxide firing	995	1:17	100	2:00	450	994
Opaquer firing	950	6:00	100	2:00	450	949
Dentin/body firing	935	4:00	60	1:00	450	934
Enamel/incisal firing	920	4:00	60	1:00	450	919
First and second correction firing	920	4:00	60	1:00	450	919
Glaze firing	910	4:00	60	1:00	450	909

T: firing temperature, S: closing time (minutes), t↑: heating rate, H: holding time, V_1_: vacuum on temperature, V_2_: vacuum off temperature

### 2.5. Marginal gap measurement

**Fig 4:**
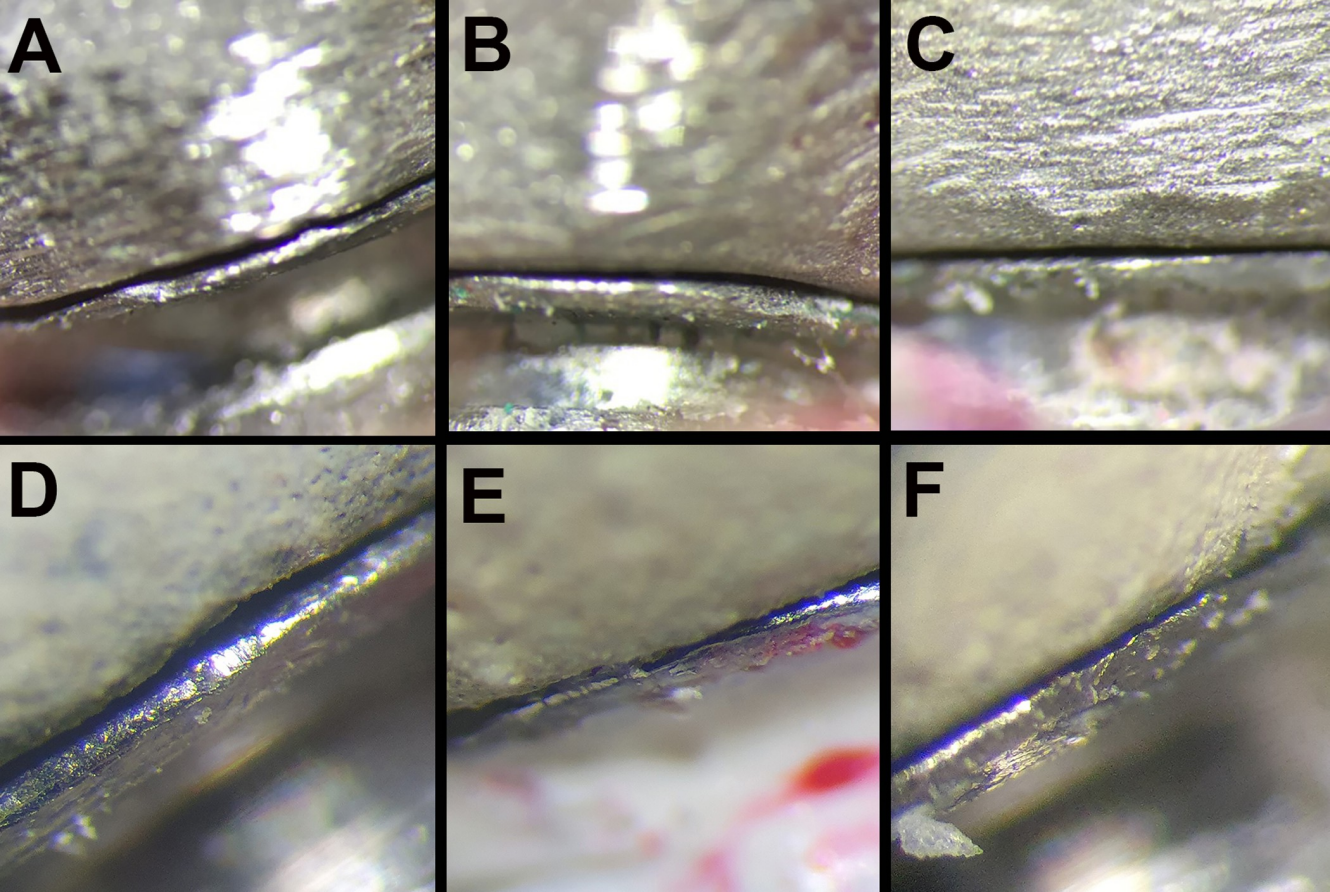
Measurement of vertical marginal gap at ×80 magnification before and after veneering: A) conventional, B) milling, C) 3D printing, and after veneering in D) conventional E) milling F) 3D printing groups.

To minimize bias during measurements, the researcher measuring the marginal gap was blinded to the specimen fabrication method and firing stage. A coding system was used to anonymously label samples. The restorations were placed on a master die and the assembly was mounted on a holding device (ensuring replication of the position by the cone-shaped tip of the holding device) and subjected to a consistent load of 15 N. To measure the vertical marginal discrepancy, a digital microscope (BUC2-500C Microscope Digital Camera with CMOS Sensor; BestScope, Beijing, China), connected to a computer and set at ×80 magnification, was used to capture images of 16 marked points (four on each of the mesial, buccal, distal, and lingual surfaces of each abutment; 32 points per sample) (**Fig 4**). Vertical misfits in each group were identified using image analysis software (ImageScope 9; Leica Biosystems, United States), calibrated by remeasuring the known distance of 1 mm at ×80 magnification, after assessing each sample. Three copings from each group were randomly selected and measurements were repeated at two-week intervals. Intra-rater reliability was assessed by calculating the intraclass correlation coefficient between the two sets of measurements, which was found to be 0.82.

### 2.6. Statistical analysis

Data analysis was conducted using SPSS software (version 20; SPSS Inc., Chicago, Illinois, USA). Mean and standard deviations of the vertical marginal gap were calculated (μm) in each group. Normal distribution and equality of variances were assessed using the Shapiro-Wilk and Levene’s test, respectively. Two-way ANOVA and Tukey’s post hoc test were used for inter-group comparisons (α = 0.05).

## 3. Results

The normal distribution and homogeneity of variance were confirmed by Shapiro-Wilk and Levene’s tests, respectively. Two-way ANOVA revealed no significant interaction between repeated porcelain firings and fabrication techniques (*P* = 0.076, Table 2). The means and standard deviations of vertical marginal gap values for the three fabrication groups at each fabrication stage are presented in Table 3.

**Table 2:** The results of two-way ANOVA for comparing the effects of fabrication method and number of firings on marginal discrepancy.

Variable	Type III Sum of Squares	df	Mean Square	F	Sig.
Fabrication method	17747.055	2	8873.528	50.438	.000
Number of firings	1500.598	2	750.299	4.265	.017
Fabrication method × number of firings	1547.185	4	386.796	2.199	.076

**Table 3:** The mean and standard deviations of vertical marginal gap values of the three fabrication groups in different stages.

Fabrication stage Method	Before veneering	After veneering		
		3 firings	5 firings	7 firings
Conventional	105.87 ± 7.23^Aa^*	110.4 ± 22.34^Aa^	102.73 ± 9.18^Aa^	101.87 ± 13.12^Aa^
Milling	77.35 ± 5.21^Aa^	115.28 ± 21.09^Ab^	96.98 ± 11.08^Aa^	94.71 ± 11.13^Aa^
3D printing	60.5 ± 3.05^Ba^	72.94 ± 7.57^Ba^	72.92 ± 6.55^Ba^	76.03 ± 5.93^Ba^

* In each column, different uppercase letters indicate significant differences; and in each row, different lowercase letters show significant differences.

The 3D printing group exhibited a significantly lower marginal gap compared to both the milling (*P*<0.001) and conventional hand-waxing (*P*<0.001) groups before the porcelain application and after each firing cycle. The milling group showed a smaller marginal gap compared to the conventional group, although this difference was not statistically significant (*P* = 0.489).

**Fig 5:**
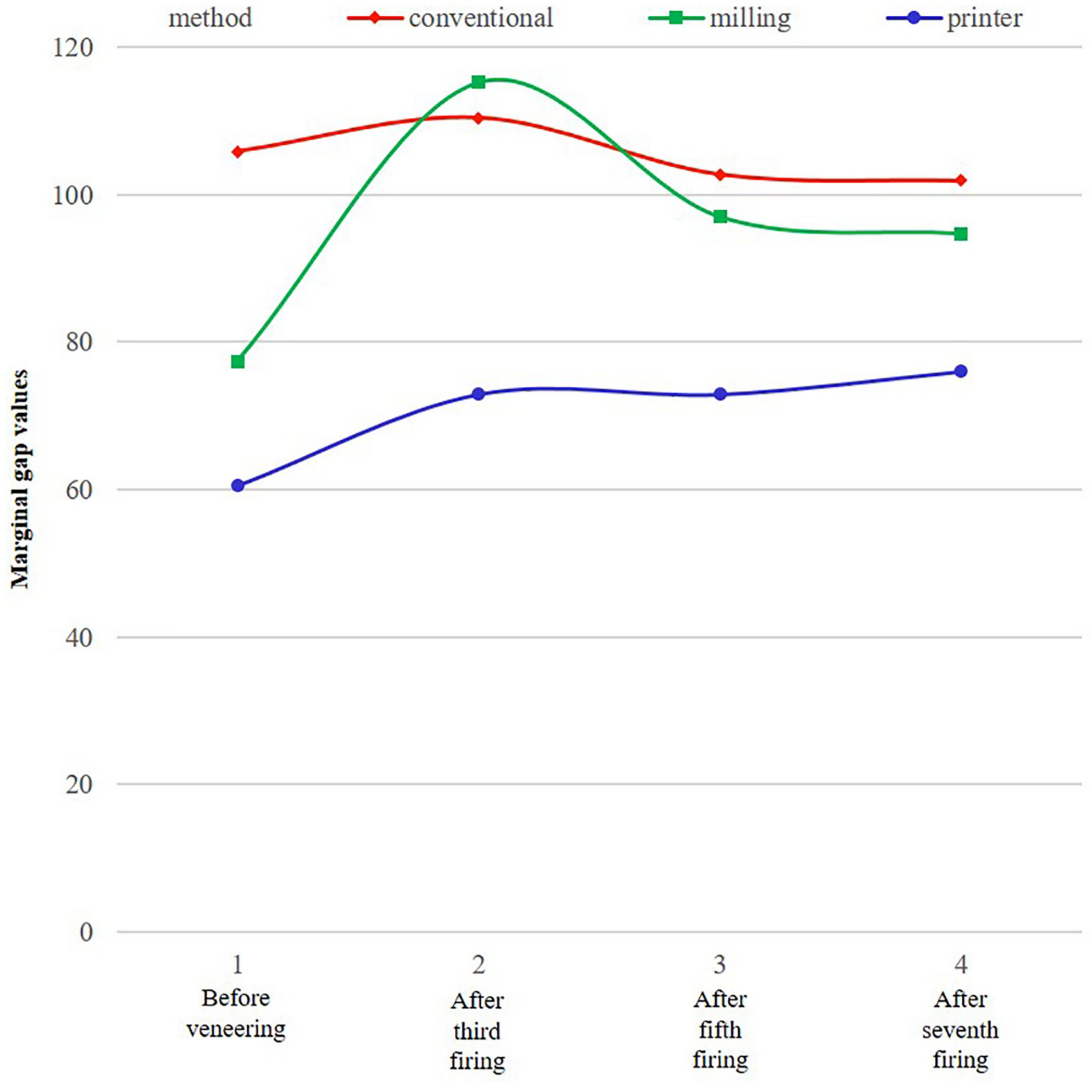
Comparison of vertical marginal discrepancy among the three fabrication methods at four different fabrication stages.

The marginal gap increased in all groups after the third (body) firing, but decreased after the fifth and seventh firings in the conventional and milling groups. However, in the 3D printing group, the marginal gap persisted with a slight further increase after the seventh firings (**Fig 5**). Marginal gap changes before and after repeated firing cycles were not statistically significant in the conventional (*P* = 0.429) and 3D printing groups (*P* = 0.501). However, the marginal gap increased significantly after 3 firings in the milling group (*P*<0.001). Additionally, this value after 3 firings was also significantly greater than that after 5 (*P* = 0.031) and 7 firings (*P* = 0.014).

## 4. Discussion

The first null hypothesis, which stated that the wax pattern fabrication method has no significant effect on the marginal adaptation of frameworks, was rejected because the 3D Printing group provided significantly superior marginal adaptation compared to the milling and conventional hand-waxing groups at all tested stages. The conventional hand-waxing technique yielded the lowest marginal adaptation; however, its difference from the milling group was not statistically significant. While additive manufacturing is an emerging field in prosthodontics, comparisons are often challenging due to variations in hardware, software, and printing parameters, which hinder standardization and attaining consistent results [30]. Nevertheless, these findings align with previous literature, which generally demonstrates superior marginal fit for CAD-CAM techniques over conventional hand-waxing [27, 37–41]. Kaleli and Saraç [40] found that additive CAD-CAM methods (direct metal laser sintering and direct process powder-bed) produced better marginal adaptation compared to the milling or conventional techniques. Kocaağaoğlu et al. [38] achieved better marginal adaptation with the laser sintering and milling methods compared to the conventional technique.

The higher vertical marginal fit observed with the CAD-CAM fabrication methods in this study may relate to the fewer production steps and less technician-dependency compared to hand-waxing [18]. Conventional fabrication involves multiple error-prone stages that rely on the technician’s skill, whereas digital workflows allow the standardization of design parameters for more consistent results [27, 51]. Furthermore, the additive manufacturing approach allows layers to be precisely shaped without limitations from overheating during milling or cutting tool size. This prevents internal stresses and enables fabrication of more detailed patterns, thereby improves adaptation over the subtractive milling technique [29]. However, some studies have found castings or subtractive methods to be comparable or even superior to additive manufacturing methods [9, 20, 42, 43]. Factors like printed layer thickness, printer models, and scanning/design/processing errors can still impact the quality of CAD-CAM additive method [28, 60], potentially contributing to the conflicting results.

In all study groups, the vertical marginal discrepancy increased after the 3^rd^ porcelain firing, but remained relatively stable thereafter. The changes were not statistically significant in the conventional hand-waxing and 3D printing groups. However, in the milling group, the marginal gap increased significantly after the 3^rd^ firing compared to the initial stage and subsequent firings. Thus, the null hypothesis of no marginal fit changes after porcelain firing was partially rejected.

Reports are conflicting regarding the effect of porcelain firing on marginal fit [5, 9, 37–39, 41–44, 51, 54, 55]. Many studies have found that ceramic firing increased gap values, especially after initial degassing or subsequent opaque firing [5, 19, 35, 36, 40, 43–51, 54–58]. However, some studies showed no significant changes [37–39, 52, 53, 59], or even marginal fit improvement [41]. Porcelain firing impacts on the marginal fit has been found to depend on the restoration fabrication method. Onöral et al. [42] found that repeated porcelain firings only did not significantly affect the marginal fit of the conventional group. Moreover, Ogunc and Avcu [9] observed that porcelain firing significantly increased the marginal gap in the milling groups, contrasting with the selective laser sintering and conventional casting groups, which supports the results of the current study. Conflicting findings in the literature can be justified by differences in materials, margin design, framework design and span, coping thickness, the employed software, firing numbers and protocols, method and number of measurements, standardization, technician skill, and research type (*in-vivo* or *in-vitro*) [9, 40].

High-temperature porcelain firing cycles can alter the alloy’s metallurgical structure [39]. The higher temperatures and faster heating/cooling rates during initial porcelain firing stages, as compared to later firings (**Table 1**), can lead to larger shrinkage and higher residual thermal stress in the veneering porcelain. These factors might have contributed to the increased vertical marginal gap observed after the third firing cycle [3, 24, 61, 62]. In addition, the milling process imparts higher residual stresses in the copings compared to the additive manufacturing and conventional wax-up techniques. This is due to factors such as high feed rates, cutting speeds, and vibration [27, 29, 58]. These intrinsic stresses were likely relieved during initial firings, contributing to the significant marginal gap increase observed in the milling group [35, 58]. Inner coping surface contamination with porcelain could have also caused misfit [35, 48, 56]. According to Zeng et al. [39], the lack of significant changes in the conventional and 3D printing groups might be due to the coping’s margin integrity, which remains intact during repeated firings.

The marginal accuracy of restorations remians a subject of debate in the literature. However, many studies have considered a maximum gap of 120 μm to be acceptable [13, 15–18]. In this study, all fabrication methods produced frameworks with vertical marginal gaps below this threshold throughout the firing cycles, indicating a clinically acceptable marginal fit.

Among the available techniques for measuring the marginal discrepancy of dental restorations, namely cone-beam computed tomography, light-body silicone weight, 3D measurement, and silicone-replica thickness [38], the direct microscopic method was selected for its simplicity, speed, and repeatability [40]. However, this method cannot assess the horizontal marginal gap [17]. Standardization was achieved by using a custom mold to ensure uniform veneering ceramic thickness, as well as performing all experiments by a single experienced dental technician. Furthermore, not cementing the copings precluded some contributing variables such as cement viscosity and seating pressures [40, 61]. A holding device was employed to ensure consistent coping seating on the die throughout the measurements.

This in-vitro study had limitations in simulating the complex intraoral environment. The absence of factors like saliva, occlusal forces, cementation effects, and thermal fluctuations means findings may not fully translate clinically. Therefore, clinical studies are essential to validate these in-vitro results under in-vivo conditions. Moreover, the direct microscopic technique used in this study could only assess the vertical marginal discrepancy. Future studies are recommended to employ 3D measurement methods for a more comprehensive fit evaluation. Additionally, further research is needed to examine multiple alloy types and long-span fixed partial dentures.

## 5. Conclusions

Within the limitations of this study, it can be concluded that:

1. The CAD-CAM 3D printing patterns resulted in significantly lower mean vertical marginal gap values compared to CAD-CAM milling and conventional hand-waxing techniques before and after porcelain firings.
2. While the milling group showed significantly increased vertical marginal opening after the third firing, the conventional and 3D printing groups exhibited no significant changes after multiple repeated firings.
3. All pattern fabrication methods achieved clinically acceptable vertical marginal fit throughout firing cycles. This confirms the ability of CAD-CAM fabricated restorations to resist firing distortions. Additive manufacturing appears promising for precise and predictable fabrication of metal-ceramic implant-supported prostheses.

## Supporting information

S1 Table Raw data for all the study groups. (XLSX)
